# Early and Late Outcome of Premature Newborns with History of Neonatal Intensive Care Units Admission at 6 Years Old in Zanjan, Northwestern Iran

**Published:** 2016

**Authors:** Mansour SADEGHZADEH, Parisa KHOSHNEVISASL, Mehdi PARVANEH, Noreddin MOUSAVINASAB

**Affiliations:** 1 Zanjan Metabolic Disease Research Center, Zanjan University of Medical Sciences, Zanjan, Iran.; 2 Social Determinants of Health Research Center, Zanjan University of Medical Sciences, Zanjan, Iran.; 3 Pediatrician, Zanjan University of Medical Sciences, Zanjan, Iran; 4 Department of Epidemiology, Zanjan University of Medical Sciences, Zanjan, Iran

**Keywords:** Mortality, NICU, Outcome, Preterm infants, Prematurity

## Abstract

**Objective:**

Premature birth is an important factor for mortality and morbidity of neonates. This study was designed to evaluate the outcome of preterm neonates who needed neonatal intensive care (NICU) hospitalization after 6 yr at their entrance to the school.

**Materials & Methods:**

This cross sectional study was conducted on premature neonates consecutively hospitalized in NICU of Valie Asr Hospital (the Academic Pediatric Hospital, Zanjan, Northwestern Iran) from September 2001 to September 2003. All children with a history of prematurity and NICU treatment were evaluated at their entrance to the school. Demographic findings, clinical examinations, IQ test, hearing and visual acuity exams were recorded.

**Results:**

From 179 neonates, 78 (43.6%) survived and were discharged from hospital. Fifty-four of them were available and entered first grade in primary school. Only one case had severe mental retardation. One case had severe retinopathy of prematurity (ROP). Hearing abnormality was not detected in any case. There was no significant relation between IQ score, visual as well as hearing findings and gestational age.

**Conclusion:**

We did not find significant disability in the outcome of surviving infants. This could be explained by the high mortality rate of neonates during hospitalization.

## Introduction

Prematurity is defined as a delivery before 37 wk of gestational age (1). The incidence of prematurity has been increased in the USA from 10.6% in 1990 to 12.8% in 2006 (2). Advances in prenatal, obstetric and neonatal care in Neonatal Intensive Care Units (NICU) were responsible for increasing preterm survivors in the last decades (3). Although the mortality rate decreased from 14.3% in 2000 to 12.4% in 2009 (4), it seems that it is associated with increased neonatal morbidity and poor neurodevelopmental outcomes (4-6). On the other hand, prematurity was the most common risk factor in infants with motor developmental delay (7).

The neurodevelopmental outcome is a very important index to assess successful treatment in the NICU (3). Although cerebral palsy, mental retardation, blindness and deafness are reported in survived preterm children, more subtle complications such as cognitive, sensory, language, visual –perceptual, attention and learning deficits are highlighted (8). The cognitive deficiency depends on biological (antenatal and postnatal) events as well as environmental factors such as socioeconomic status, parental occupation and education, number of siblings, and breastfeeding (9). Although the prevalence of cognitive dysfunction was similar in different cultural environments, it is well recommended to evaluate the cognitive function of these children in a long-term follow up in different populations (10). Some investigations had studied the outcome at 2 yr of age (11), but other surveys have followed the children at the time of school with the conception that longer periods of follow up reveal more disabilities (12). The follow up of these children will improve our knowledge of preterm brain development and treatment strategies (8).

Neonatal morbidities and outcome are different in literature, which could be the result of a discrepancy in sample size or methods of studies, lack of control groups, different inclusion criteria, differences in severity determination and methods of treatment (5, 9). 

Regarding that similar studies have not been performed in our region, we conducted this study to evaluate the early and late outcome of neonates admitted in NICU at their entrance to the school.

## Materials & Methods

The aim of this study was to define the early and late outcome of premature infants surviving the NICU at their entrance to the school. This descriptive study was conducted on premature neonates consecutively hospitalized in NICU of Valie Asr Hospital (academic pediatric hospital) in Zanjan, northwestern Iran from September 2001 to September 2003. All these children were evaluated at their entrance to school, when they were six yr old (2007-2009).

This study was approved by the Ethical Committee of Zanjan University of Medical Sciences.

Parental informed consent was taken prior to enrollment in the study. The children were assessed by a pediatrician, physically examined, and the history of any underlying disease or taking any medication was investigated. The children’s intelligent quotients (IQ) were determined by Wechsler test. The IQ more than 84 was considered normal. For each child audiometry was performed by a trained audiologist and visual acuity was investigated by an optometrist using Snellen charts.

Meanwhile, their hospital records were evaluated retrospectively. All data, including mortality rate, gestational age, birth weight, sex, Apgar score, history of neonatal diseases, and diagnostic procedures such as intracranial ultrasonography, CT scan and need for ventilators were inserted in prepared questionnaires. 

All collected data were analyzed by SPSS software version 16.0 (Chicago, IL, USA). Comparisons for categorical variables were performed by chi-square test. P value less than 0.05 was considered statistically significant.

## Results

Overall, of 179 neonates, 101 (56.4%) cases died because of some of common causes of death including hyaline membrane disease or HMD (37.5%), HMD and sepsis (9%), asphyxia (4%), and disseminated intravascular coagulation with IVH (1.7%). 

The mortality rate in neonates with a gestational age of 28-34 wk was 50%. There was a significant relation between gestational age and mortality rate (P= 0.001).

Seventy-eight (43.6%) newborns survived and enrolled in the study, of them, 53 newborns (68%) had a gestational age of 28-34 wk and 25 (32%) patients had a gestational age of 34-37 wk. Among them, 24 (31%) were female.

The neonatal birth weights varied between 450 gr to 3000 gr with the mean of 1586.21 ±540.53 gr. First minute Apgar scores were less than7 in 21 (27%), and 7 or more in 57 (73%) cases.

There was a significant relationship between first minute Apgar score <7 and neonatal mortality.

(P=0.000). Intracranial ultrasonography was normal in survived patients and approved by brain CT scans. Four (5%) patients were assisted by ventilator. Frequency of neonatal diseases and their mortality have shown in Table 1.There was no significant relationship between mortality rate and some variables, including gender, weight, ventilator assisting, imaging findings and neonatal diseases.

The survived neonates were followed at their entrance to school from 2007 to 2009. Twenty-four children were excluded due to immigration, lack of full URL in the file and the lack of information about their new residence.

Finally, 44 patients were evaluated by physical examination, audiometric and optometric assessment and IQ test. None of them had chronic disease, seizure or history of medications.

The audiometric assessment was normal in all of the patients. One of the patients with visual problems had been consulted with an ophthalmologist. Because of retinopathy of prematurity, he was treated with laser therapy. Fifty-one patients (94.4%) had normal IQ and three patients (5.6%) showed mental retardation. 

One patient had mild, one moderate and the other had severe mental retardation. Two patients with abnormal IQ were in the group of infants with gestational age of 28-33 wk and one patient with abnormal IQ was found in infants with gestational age of 33-37 wk (Table 2). 

Even though the percentage of the lower IQ was found in the more mature infants, these differences were not statistically significant.

There was not statistically significant relation between GA with IQ, hearing and visual abnormality.

## Discussion

The numbers of premature live births are increasing, associated with increased morbidity and mortality (13, 14). In this study, the mortality of 179 premature neonates in NICU was 56.4% compared to 64. 4% in a previous study in Iran (15), 80.8% in Alexandria (1) and 86% in Saudi Arabia (16). Although, based on the Vermont Oxford network multicentric study in 33 North American (VON) centers, the mortality rate was 1.4% (11). The mortality of premature neonates weighing 501-1500 gr born in the USA from 2000 to 2009 decreased from 14.3% to 12.9% (4). These differences could be the result of discrepancies in gestational ages and treatment facilities in different studies. 

In our study, 54% of survived infants were male and there was no significant difference in mortality rate of two genders. These findings are similar to a study in Turkey (17).

In the present study, there was a significant relationship between gestational age and mortality rate, similar to some other studies (1, 15, 16). The decrease in mortality of tiny neonates (501-750 g) was more than other groups (4). Another study had similar results with a 4.6% decrease in mortality of those premature infants (18). Difference in our results could be due to a lesser prenatal care and maternal factors such as malnutrition and lower treatment facilities in different hospitals. 

Our study showed a statistically significant relationship between mortality rate and low first minute Apgar score, similar to previous studies (15, 16). Another study showed that 75% of neonates with a 10 min Apgar score of 0-3 died or suffered a disability compared to 45% of neonates with Apgar scores more than three and increscent in Apgar score was significantly related to a better outcome (19).

We found Hyaline membrane disease (HMD) in 95.3% of our patients, similar to other studies (15, 20). In 38.9% of patients, we found associated complications with HMD that the most frequent was sepsis (18.5%). 

In previous studies (14, 15, 20), this association was 43.6%, 30.9% and 76%, respectively.

The type of deliveries in our study was through cesarean section at (43%), but previous studies revealed that 59% and 15% of neonates were delivered by this method respectively (2, 16).

Premature rupture of membranes was seen in 10.1% of our neonates similar to another study as 13.4% (2).

Intraventricular hemorrhage (IVH) was found in three patients. This complication is reported in 3.5% of neonates (14), besides 8.5% of the evaluated patients had severe IVH (11). Fourteen percent of patients showed IVH grade > II (17). IVH was found in 16% and 9.5% in preterm neonates (13, 21). The lower rate of IVH in our study may be due to the shorter duration of survival in our very low birth weight neonates. Neonates suffering IVH had poorer outcomes (22).

Therefore, better outcome of our patients may be related to lesser IVH in our survived neonates. The weight of 29.6% of our patients was below 5th percentile at school entrance. SGA infants had suboptimal growth until age 5 in Finland (10).

Comparing late preterm and term infants, preterm infants are at increased risk of underweight and stunting (23).

Visual disturbance due to retinopathy of prematurity (ROP) was diagnosed in 1.9% of our patients. Severe ROP stage 3–4 was found in 20.9% of the evaluated patients with 1.2% bilateral blindness (11) and 10.3% with one blindness (21). Seven percent of neonates showed severe ROP (13). The lesser rate of visual disturbance may be due to poorer outcome of our neonates.

The auditory assessment of our neonates revealed no abnormality, but the study of Mercier et al. on extremely low birth weight infants showed 1.9% of hearing loss (11). In previous studies, three and one patient (s) was deaf (21, 24). This difference may be explained by the fact that in these surveys only neonates less than 28 weeks of gestational age have been studied. 

In our study, 94.4% of patients had normal IQ with one patient having severe mental retardation. There was not a significant relationship between IQ and gestational age. This finding is very similar to a study in Nepal (25) but is different from the results of some other studies (6, 20, 26-30). Minor and major impairment in school performance was diagnosed in 39% and 18% in preterm infants respectively and the disability had an inverse relation with gestational age (12). A relation was found between lower neurodevelopmental outcomes with low Apgar score, gestational age less than 37 weeks and neonatal respiratory problems at one year of age (30). 

The IQ assessment in preschool age showed that the mean IQ level in 89% of less than 28 wk of gestational age was 94 ± 15 (24). A limited association between gestational age and cognitive function was found in this study. These differences could be explained by the fact that in our study only patients with higher gestational age had a chance to survive.

The differences in early and late neonatal outcome could be the result of a discrepancy in inclusion criteria, differences in severity determination, methods of treatment and rehabilitation therapies.

**In conclusion, **despite the increase in diagnostic and treatment facilities, the neonatal mortality, particularly in preterm infants remains a serious problem, especially in developing countries. Factors such as gestational age, low birth weight, low Apgar score in the first minute and associated complications in these neonates were effective in their outcome but gender did not play a role in this field.

Since most infants discharged alive in our study had higher gestational age and birth weight, their evaluation at school entering age has been associated with the minimum expected disturbances.

**Table 1:** Neonatal Diseases in Survived Cases

**Variable**	**Number(%)**
**HMD**	47 (60)
**Asphyxia**	1 ( 1.3)
**Sepsis**	2 (2.6)
**HMD+ Sepsis**	17(21.8)
**Hypoglycemia**	5 (6.5)
**NEC**	1 (1.3)
**GIB**	1 (1.3)
**Apnea**	4 (5.2)

HMD =hylaine membrane disease, NEC=Necrotizing Entrocolitis; GIB=Gastrointestinal Bleeding

**Table 2:** Relation between Gestational Age and IQ, Auditory and Visual Abnormality

**Variables**		**Gestational Age(week)** **Number(%)**		**Total**	**P Value**
		**28 - 33**	**34 -37**		
**IQ**	**Normal**	36	15	51	NS
	**Abnormal**	2	1	3	
	**Total**	38	16	54	
**Auditory**	**Normal**	38	16	54	NS
	**Abnormal**	-	-	-	
	**Total**	38	16	54	
**Visual**	**Normal**	37	16	53	NS
	**Abnormal**	-	-	1	
	**Total**	38	16	54	
